# Cost‐Effectiveness Analysis of Adjuvant Alectinib versus Platinum‐Based Chemotherapy in Resected ALK‐Positive Non‐Small‐Cell Lung Cancer in the Chinese Health Care System

**DOI:** 10.1002/cam4.70405

**Published:** 2024-11-18

**Authors:** Qiran Wei, Yifang Liang, Jiahui Mao, Xin Guan

**Affiliations:** ^1^ School of International Pharmaceutical Business, China Pharmaceutical University Nanjing Jiangsu Province China; ^2^ Center for Pharmacoeconomics and Outcomes Research of China Pharmaceutical University Nanjing Jiangsu Province China

**Keywords:** adjuvant treatment, alectinib, China, cost‐effectiveness analysis, platinum‐based chemotherapy

## Abstract

**Objectives:**

The ALINE trial demonstrated the superiority of alectinib over platinum‐based chemotherapy in resected Anaplastic Lymphoma Kinase (ALK)‐positive non‐small‐cell lung cancer (NSCLC). Considering the high cost of alectinib, this study aimed to evaluate the economic value of alectinib compared to platinum‐based chemotherapy for treating early‐stage ALK‐positive NSCLC from the perspective of the Chinese health care system.

**Materials and Methods:**

We developed a five‐state Markov model with monthly cycles to estimate the lifetime costs, life‐years (LYs), quality‐adjusted life‐years (QALYs), and incremental cost‐effectiveness ratios (ICERs) in terms of cost per LY gained and per QALY gained. Costs were obtained from database, expert opinions and published literature, and utilities were primarily derived from a multicenter cross‐sectional study based on the Chinese population. Costs and outcomes were discounted at 5% per year. Sensitivity analyses and scenario analyses were conducted to assess uncertainty in model results.

**Results:**

Compared to platinum‐based chemotherapy, alectinib increased total costs by $16,245 and provided gains of 2.02 LYs and 1.84 QALYs over a lifetime horizon. ICERs were $8,052/LY and $8,806/QALY. The ICER in terms of cost per QALY gained was most sensitive to the outcome discount rate. Probabilistic sensitivity analysis indicated a 93% probability of alectinib being cost‐effective at a willing‐to pay (WTP) threshold of $12,367/QALY (1 GDP per capita), rising to 100% at $37,100/QALY (3 GDP per capita).

**Conclusion:**

Alectinib appears to be the preferred cost‐effective option in the adjuvant treatment for Chinese patients with resected early‐stage ALK‐positive NSCLC.

## 1 Introduction

Lung cancer has the highest incidence and mortality rates among all malignant tumors worldwide. In 2022, there were 1.06 million new cases of lung cancer, accounting for 22.0% of all malignant tumors, and 733,300 deaths, accounting for 28.5% of all malignant tumor‐related deaths worldwide [1]. Non‐small‐cell lung cancer (NSCLC) accounts for approximately 85% of lung cancer patients, with anaplastic lymphoma kinase (ALK) rearrangements occurring in approximately 3%–7% of NSCLC patients [2].

While surgical resection is the preferred treatment for early‐stage NSCLC [3], patients undergoing this procedure still face risks of postoperative recurrence and mortality [4]. Adjuvant chemotherapy has emerged as a rational treatment strategy to reduce residual lesions, thus lowering the risk of recurrence and improving patients' quality of life [5]. Platinum‐based adjuvant chemotherapy is the standard treatment for resected stage II or III NSCLC patients. The addition of platinum‐based adjuvant chemotherapy has been shown to increase the five‐year overall survival (OS) rate by approximately 4%–5.4% and the five‐year disease‐free survival (DFS) rate by approximately 5.8% [6, 7, 8, 9]. However, adjuvant chemotherapy is often associated with severe adverse events (AEs) and toxicity, leading to high discontinuation rates and suboptimal completion rates, with limited clinical benefits [10, 11].

Alectinib is a type of ALK tyrosine kinase inhibitor which is recommended as a first‐line (1 L) treatment for ALK‐positive patients by European and Chinese guidelines [12, 13]. Its indication for the treatment of advanced NSCLC has been included in the Chinese national reimbursement drug list (NRDL). Moreover, the recent approval of alectinib by the Chinese National Medical Products Administration for adjuvant treatment in resected ALK‐positive NSCLC patients offers a promising therapeutic option. The latest published ALINE trial demonstrated significant benefits of alectinib in this setting, including prolonged DFS, reduced risk of disease recurrence, prevention of brain metastases, and a favorable safety profile [14]. However, there is currently a lack of economic studies regarding its usage in early‐stage ALK‐positive NSCLC. Considering the high cost of alectinib, such analyses are crucial for informing treatment decisions and resource allocation within the Chinese healthcare system. Therefore, this study aimed to evaluate the economic value of alectinib compared to platinum‐based chemotherapy for the treatment of early‐stage ALK‐positive NSCLC from the perspective of the Chinese health care system.

## 2 Methods

This study followed the Consolidated Health Economic Evaluation Reporting Standards reporting guideline [15] (Table S1). Key assumptions and model inputs were discussed and validated with five clinical experts specialized in lung cancer respectively.

### 2.1 Model Overview

A Markov model was constructed using Microsoft Excel in an academic medical setting to simulate the lifetime outcomes of alectinib versus platinum‐based chemotherapy as adjuvant treatment in patients with early‐stage ALK‐positive NSCLC. A lifetime horizon was used in the base‐case analyses to capture overall outcomes. The cycle length was set at one month, and half‐cycle corrections were applied in the model, assuming that transitions across health states occur in the middle of each cycle. Model outcomes included total costs, life‐years (LYs), quality‐adjusted life‐years (QALYs), and incremental cost‐effectiveness ratios (ICERs) in terms of cost per LY gained and per QALY gained. Costs and outcomes were discounted at 5% per year according to China guidelines for pharmacoeconomic evaluations [16]. For this study, the perspective of Chinese health care system was adopted.

**FIGURE 1 |:**
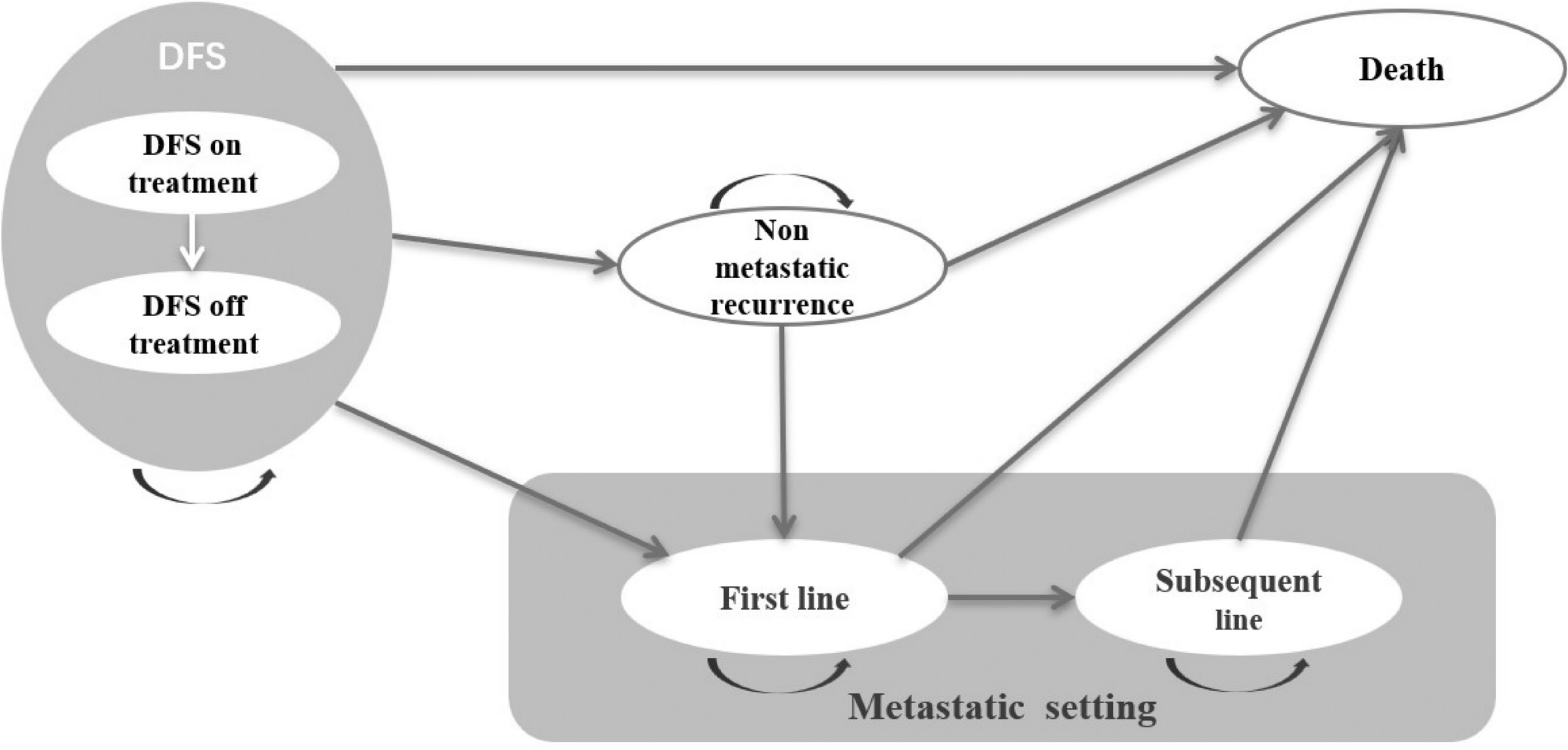
Model structure. DFS, disease‐free survival.

The Markov model included five health states: DFS, nonmetastatic recurrence (including local and regional recurrence), 1 L metastatic recurrence, subsequent line metastatic recurrence and death (see Figure 1). DFS was defined as the time from randomization to the first documented recurrence of disease or new primary NSCLC, or death from any cause. In the DFS health state, patients received either oral alectinib at a dose of 600 mg twice daily or intravenous platinum‐based chemotherapy in four 21‐day cycles. Chemotherapy options included cisplatin at a dose of 75 mg per square meter of body surface area on day 1 of each cycle, along with vinorelbine at a dose of 25 mg per square meter on days 1 and 8, gemcitabine at a dose of 1250 mg per square meter on days 1 and 8, or pemetrexed at a dose of 500 mg per square meter on day 1. All patients were considered to be in the DFS health state until they experienced disease progression or death. Patients could transition from the DFS health state to the nonmetastatic recurrence state or 1 L metastatic recurrence state.

Expert interviews were conducted with lung cancer experts to understand the proportion of patients receiving active treatment and the treatment regimens used following metastatic recurrence. In the locoregional recurrence state, 83.9% of patients underwent active treatment, while the remaining patients received supportive care only. For 1 L metastatic recurrence, 90.9% received active treatment, and for 2 L metastatic recurrence, 87.5% received active treatment, with the remaining patients receiving supportive care. According to clinical guidelines [12] and expert opinions, active treatment included radiotherapy and six cycles of chemotherapy (platinum‐based + paclitaxel). For patients with 1 L metastatic recurrence, 50% received lorlatinib, while the other 50% were treated with alectinib. For those experiencing subsequent line metastatic recurrence, 36.3% of patients received alectinib, 27.2% received brigatinib, and 36.3% received platinum‐based doublet chemotherapy plus bevacizumab with or without immunotherapy [18]. It was assumed that patients actively receiving treatment progressed to disease metastasis rather than death due to the disease when in the nonmetastatic recurrence and 1 L metastatic recurrence health states. However, individuals receiving supportive care can only progress to death.

### 2.2 Modeled Population

The target population information was derived from the ALINA trial. The data incorporated into the model at the time of analysis corresponded to the cut‐off date of June 26, 2023. Eligible patients were aged 18 years or older and had completely resected, histologically confirmed stage, II, or IIIA NSCLC. According to the ALINA trial, the mean body weight of the alectinib group was set at 68.33 kg, with an average body surface area of 1.85 m^2^; while the mean body weight of the chemotherapy group was set at 70.96 kg, with an average body surface area of 1.88 m^2^.

### 2.3 Clinical Efficacy

**FIGURE 2 |:**
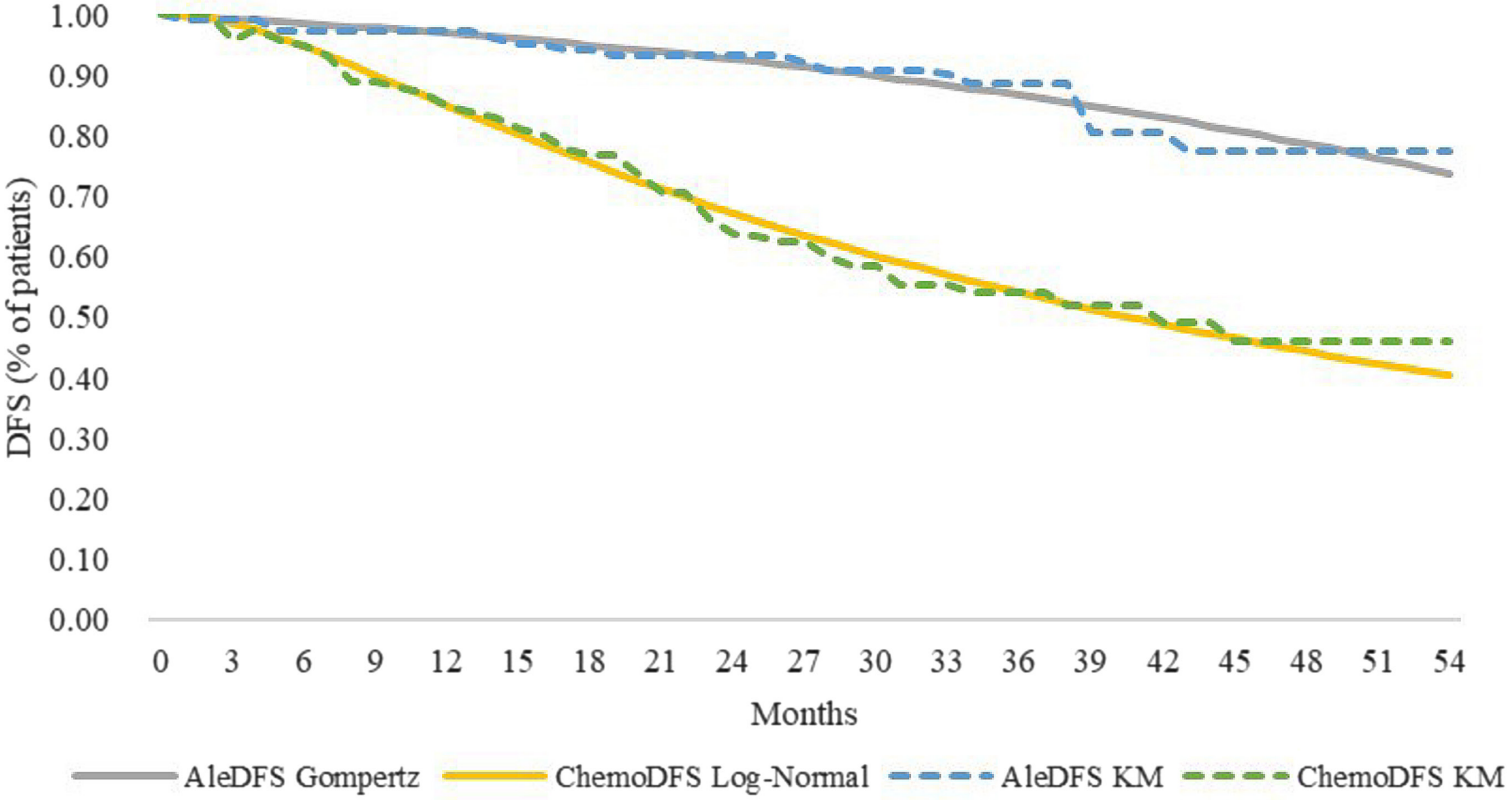
Kaplan–Meier curves and parametric survival distributions for DFS. Ale, alectinib; Chemo, chemotherapy; DFS, disease‐free survival.

The survival probabilities for DFS health state were based on the ALINA trial. The survival and progression probabilities for metastatic recurrence health state were based on the CROWN trial for lorlatinib [20] and a real‐world study for alectinib [21]. Extrapolation was necessary in the cost‐effectiveness analysis due to the limited follow‐up time of the Kaplan–Meier (KM) curves in clinical trials. To accurately capture the value of treatment regimens in extrapolation, we considered standard parametric model and Royston and Parmar (RP) spline model. Since cure is clinically plausible for the early‐stage NSCLC population and the obvious plateau at the end of the DFS curves, the mixture cure model was also considered [22]. The standard parametric model used Generalized Gamma, Weibull, Gompertz, Loglogistic, and Log‐normal distributions. The number of knots for the RP spline model was set from three to five, with linking functions set as hazard, normal, and odds. The model selection was based on the lowest Akaike information criterion values, and visual examination verified the adequacy of the distribution fit to the KM curve. The pseudo‐individual patient data were extracted using GetData Graph Digitizer (v2.25, http://getdata.sourceforge.net/download.html) from the clinical trial and were then reconstructed and fitted by the standard parametric models using R (v3.6.0, https://www.r‐project.org/). The similar method was used to reconstruct and extrapolate OS and PFS curves. Results of fitting to the observed data and the parameter values in the ITT population are presented in Table S2. The preferred distribution of DFS curves was Gompertz for alectinib and Log‐normal for chemotherapy. The KM and parametric survival distributions for DFS health state used in the model are shown in Figure 2. The KM and parametric survival distributions for the OS and PFS curves of lorlatinib and alectinib used in the model are shown in Figure S1.

Based on the findings of Sonoda et al. [23], patients with early‐stage NSCLC who underwent complete curative resection and experience recurrence ten years later were considered to have ultra‐late recurrence, with a recurrence rate of only 2.5%. Therefore, this study assumed that patients who had not experienced recurrence were considered cured (or free of a DFS event) at 24 months, with a maximum cure rate of 97.5% at ten years. For patients who had been cured and no longer face the risk of cancer‐related death, their probability of mortality should be higher than that of individuals in the general population, assuming a 25% excess mortality rate compared to the general population [24, 25]. The results of the ALINE trial indicated that despite a treatment duration of only two years, the efficacy of alectinib exhibits sustained effects in the later follow‐up period. However, the ALINE data and existing literature lacked adequate information to pinpoint the exact time point at which the effectiveness of alectinib treatment ceases. In this study, we made a conservative assumption, positing that the efficacy of alectinib gradually declined over time, starting from the 28th month (the median duration of follow‐up for survival in ALINE), and ceased by the fifth year.

The proportion of patients with distant metastases among recurrent patients was based on the ALINE trial, revealing a rate of 35.71% in the alectinib group and 55.10% in the chemotherapy group. Transition probability from nonmetastatic recurrence to metastatic recurrence was obtained from a retrospective study [26], which were assumed to be equivalent in both groups. Age‐specific background mortality rates were obtained from China Population Census Yearbook [27]. We estimated a monthly probability of death among women under supportive care to be 0.0762 in the nonmetastatic recurrence state [28], and to be 0.1036 in the metastatic recurrence state [17].

### 2.4 Costs

We estimated the costs in 2024 US dollars, with an exchange rate of 1 USD = 7.23 Chinese Yuan inflated using the Medical Consumer Price Index (http://www.stats.gov.cn/). Only direct medical care costs were considered, as the perspective of Chinese health care system was used. These costs included drugs, administration, follow‐up, AEs, and end‐of‐life costs. The treatment costs per cycle were calculated by multiplying the unit costs of the drugs by the dosing schedules for one cycle. The cost of alectinib was based on the latest reimbursable price under Chinese medical insurance in 2024, and the costs of other drugs were based on the median price of the bid‐winning products on China drug bidding database (https://www.menet.com.cn/). The proportions of radiotherapy and chemotherapy in the nonmetastatic recurrence health state were derived from a retrospective study by Wong et al. [17], which analyzed the recurrence of 9001 surgically resected stage I‐III NSCLC patients. We employed a cost per unit of drug approach to account for unused drug waste in the model, assuming that all patients received the full prescribed doses of their assigned treatment with a consistent dose intensity. Follow‐up costs and administration costs were obtained from the health care documents across seven provinces and cities, including Beijing, Shanghai, Guangdong, Zhejiang, Hunan, Fujian, and Shaanxi. These expenses included medical services such as diagnosis, blood biochemistry tests, blood tests, urine analysis, CT scans, MRI scans, as well as injection, nursing, monitoring, and bed fees. End‐of‐life cost was extracted from research conducted by Cao et al. [29], which surveyed medical expenses for advanced cancer patients in end‐of‐life care institutions in China. We considered only severe AEs (≥ grade 3) with an incidence of ≥ 5% during adjuvant therapy. All AEs assumed to occur during the first treatment cycle. The detailed costs are listed in Tables S3, and a summary is shown in Table 1.

**TABLE 1 |:** Key inputs for the Markov model.

Model inputs	Value	Distribution	Low	High	Source
Proportion of patients with distant metastases among recurrent patients					
Ale group	0.357	BETA	0.286	0.429	ALINE trial
Chemo group	0.551	BETA	0.441	0.661	ALINE trial
Transition probabilities					
From nonmetastatic recurrence to 1 L metastatic recurrence (active treatment)	0.018	BETA	0.015	0.022	[26]
From nonmetastatic recurrence to death (supportive care)	0.076	BETA	0.061	0.091	[28]
From 1 L metastatic recurrence to death (supportive care)	0.104	BETA	0.083	0.124	[17]
From subsequent line metastatic recurrence to death (supportive care)	0.104	BETA	0.083	0.124	[17]
Direct costs per cycle					
					
Chemo in DFS	412.87	GAMMA	245.49	691.93	MENET
Follow‐up visit for 0 to 5 years	24.42	GAMMA	19.54	29.31	Health care document
Follow‐up visit after 5 years	12.21	GAMMA	9.77	14.65	Health care document
Administration	43.36	GAMMA	34.69	52.03	Health care document
Cost of treatment for nonmetastatic recurrences (Ale group)	334.95	GAMMA	267.96	401.94	MENET, [17, 43]
Cost of treatment for nonmetastatic recurrences (Chemo group)	338.15	GAMMA	270.52	405.77	MENET, [17, 43]
Cost of treatment for 1 L metastatic recurrences	1855.02	GAMMA	1484.01	2226.02	MENET, [43]
Cost of treatment for subsequent line metastatic recurrences (Ale group)	2659.42	GAMMA	2127.53	3191.30	MENET, [43]
Cost of treatment for subsequent line metastatic recurrences (Chemo group)	2719.49	GAMMA	2175.43	3263.14	MENET, [43]
End of life	1967.49	GAMMA	764.34	5254.75	[29]
Costs of AEs per cycle					
Blood creatine phosphokinase increased	0.40	GAMMA	0.32	0.48	Expert opinions, MENET
Neutrophil count decreased	17.65	GAMMA	14.12	21.18	Expert opinions, MENET
Risk of AEs					
Blood creatine phosphokinase increased	0.062	BETA	0.056	0.068	ALINE trial
Neutrophil count decreased	0.102	BETA	0.092	0.112	ALINE trial
Health state utilities (per year)					
DFS (on treatment)	0.845	BETA	0.820	0.870	Multicenter cross‐sectional study
DFS (off treatment)	0.872	BETA	0.845	0.951	Multicenter cross‐sectional study, [32]
Nonmetastatic recurrence	0.845	BETA	0.820	0.870	Multicenter cross‐sectional study
1 L metastatic recurrence	0.805	BETA	0.775	0.835	Multicenter cross‐sectional study
Subsequent line metastatic recurrence	0.741	BETA	0.703	0.779	Multicenter cross‐sectional study
Disutilities of AEs (per year)					
Blood creatine phosphokinase increased	0.062	BETA	0.045	0.093	[31]
Neutrophil count decreased	0.200	BETA	0.180	0.220	[30]
Cost discount rate	5%	CONSTANT	0%	8%	[16]
Outcome discount rate	5%	CONSTANT	0%	8%	[16]

Abbreviations: Ale, alectinib; AEs, adverse events; Chemo, chemotherapy; DFS, disease‐free survival; 1 L: first‐line.

### 2.5 Utilities

Utilities for each health state were derived from a multicenter cross‐sectional study conducted by China Pharmaceutical University and Beijing University of Chinese Medicine from June 2022 to April 2024 across 18 provinces or cities in China, involving 20 hospitals. The study, which obtained ethical approval under protocol number SECCR/2021–192‐01, employed face‐to‐face questionnaire surveys to collect EQ‐5D‐5L data from 947 patients with NSCLC. Utility decrements associated with AEs were derived from published literature [30, 31]. Increased blood creatine phosphokinase often manifested as muscle pain in patients. Due to the lack of literature reporting the disutility related to increased blood creatine phosphokinase, the disutility of pain was used instead of its disutility [31]. Utilities and disutilities of health states and AEs were listed in Table 1.

### 2.6 Sensitivity Analyses

One‐way deterministic sensitivity analyses (DSA) and probabilistic sensitivity analyses (PSA) were conducted to determine the impact of the uncertainty on the input parameters on the ICER in terms of per QALY gained. In the one‐way DSA, we examined the effect of each parameter on the ICER by adjusting parameters within their minimum and maximum values. The cost range of chemotherapy was derived from the MENET database. Discount rates of 0% and 8% were used as the upper and lower bounds. The upper limit value of DFS utility (off treatment) was derived from the general population utility data in China [32]. For other input parameters, we set the upper and lower limits based on 95% confidence interval when possible. In cases where such data were not available, we employed reasonable variations, such as ±10% for utilities and the risk of AEs, and ± 20% for other parameters around the base‐case values [33]. The results were showed in a tornado diagram.

The PSA was performed using 5000 Monte Carlo simulations. To derive parameter estimates for each simulation, gamma distributions were used for costs and beta distributions were used for utilities, risk of AEs, proportion of patients with distant metastases among recurrent patients and some transition probabilities. The study applied criteria in China guidelines for pharmacoeconomic evaluations [16] to determine cost‐effectiveness, based on a willingness‐to‐pay (WTP) threshold of 1–3 times the GDP per capita per QALY. The results were showed as acceptability curves and a cost‐effectiveness scatterplot.

### 2.7 Scenario Analyses

Three scenario analyses were conducted to further explore the influence of parameter uncertainty on the research results.

Scenario 1: For patients with 1 L metastatic recurrence, 100% received either lorlatinib or alectinib.

Scenario 2: Although the lifetime horizon can fully capture and reflect the cost and health outcome differences of different interventions, curve extrapolation may bring uncertainty. Therefore, our analysis used alternative time‐horizons (10, 20, and 30 years) to assess how costs and outcomes were affected by the shorter time horizon.

Scenario 3: Two possible ratios were determined based on expert opinions. These proportions were set at 0.9, 0.8, and 0.5 as well as 0.8, 0.6, and 0.4 for nonmetastatic recurrence, 1 L metastatic recurrence and subsequent line metastatic recurrence health states.

## 3 Results

### 3.1 Base‐Case Analyses Results

The results of the base‐case analyses are presented in Table 2. The alectinib group resulted in 11.44 LYs and 9.82 QALYs, with a cost of $75,562. The platinum‐based chemotherapy group resulted in 9.42 LYs and 7.97 QALYs, with a cost of $56,317. The proportion of LYs spent in the DFS health state was 82.95% with alectinib and 66.94% with platinum‐based chemotherapy. Compared to adjuvant chemotherapy, the upfront costs of two‐year adjuvant alectinib treatment were partly offset by reduced costs of subsequent treatment, administration, follow‐up, and terminal care. The resulting ICERs of alectinib versus chemotherapy were $8052/LY and $8806/QALY.

**TABLE 2:** Base‐case analyses results.

Results	Ale	Chemo	Difference
LYs			
LYs in DFS	9.49	6.31	3.18
LYs in nonmetastatic recurrence	0.61	0.69	−0.07
LYs in 1 L metastatic recurrence	1.18	2.05	−0.86
LYs in subsequent line metastatic recurrence	0.15	0.38	−0.23
Total LYs	11.44	9.42	2.02
QALYs			
QALYs in DFS	8.23	5.46	2.77
QALYs in nonmetastatic recurrence	0.52	0.58	−0.06
QALYs in 1 L metastatic recurrence	0.95	1.65	−0.69
QALYs in subsequent line metastatic recurrence	0.11	0.28	−0.17
Total QALYs	9.82	7.97	1.84
Costs ($)			
Costs in DFS	42,245	2514	39,731
Costs in nonmetastatic recurrence	573	675	−102
Costs in 1 L metastatic recurrence	27,167	46,989	−19,822
Costs in subsequent line metastatic recurrence	2074	5327	−3253
Costs in end‐of‐life care	502	812	−310
Total Costs	72,562	56,317	16,245
ICER ($/LY)	8052		
ICER ($/QALY)	8806		

Abbreviations: Ale, alectinib; Chemo, chemotherapy; DFS, disease‐free survival; ICER, incremental cost‐effectiveness ratio; LY, life‐year; 1 L, first‐line; QALY, quality‐adjusted life‐year.

### 3.2 Sensitivity Analyses Results

**FIGURE 3 |:**
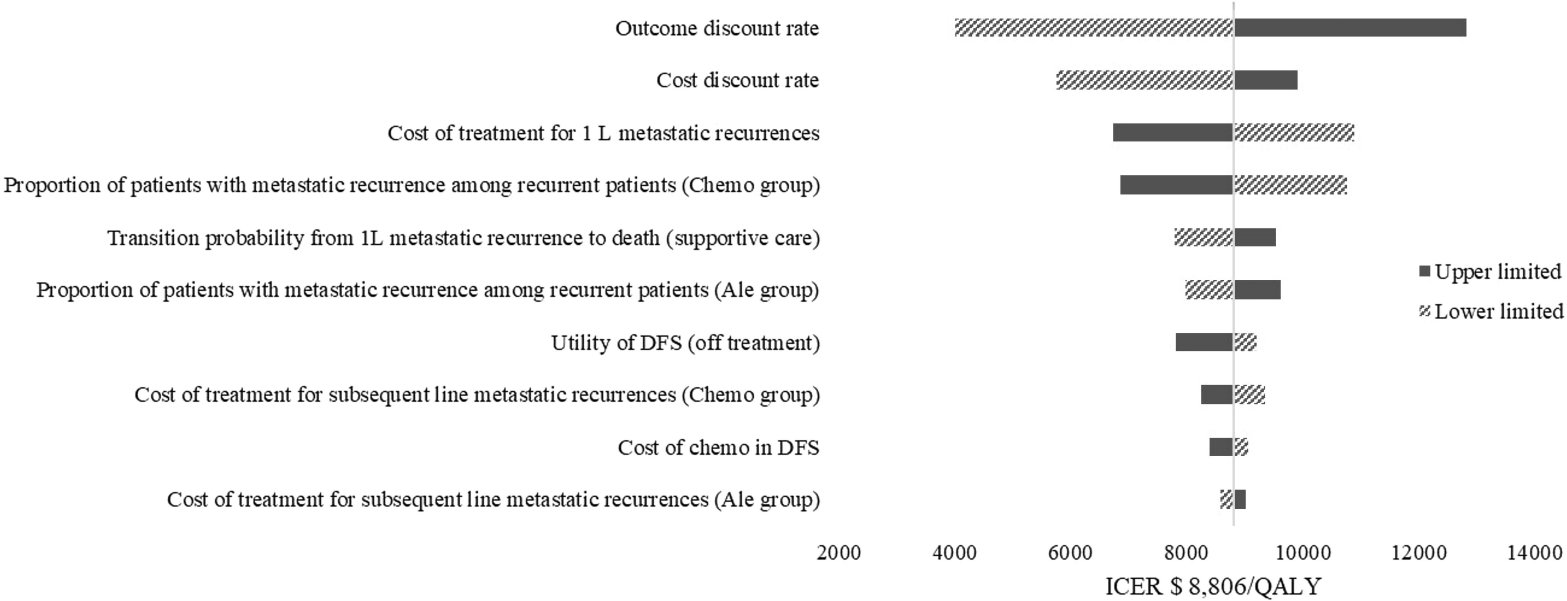
Tornado diagram. Ale, alectinib; Chemo, chemotherapy; DFS, invasive disease‐free survival; ICER, incremental cost‐effectiveness ratio; QALY, quality‐adjusted life‐year; 1 L, first‐line.

The top 10 variables that were most influential on results are shown in Figure 3. Among these, the ICERs was most sensitive to the outcome discount rate. The range of the one‐way DSA was from $4007/QALY to $12,828/QALY, indicating that the results of the base‐case analyses were relatively stable.

**FIGURE 4 |:**
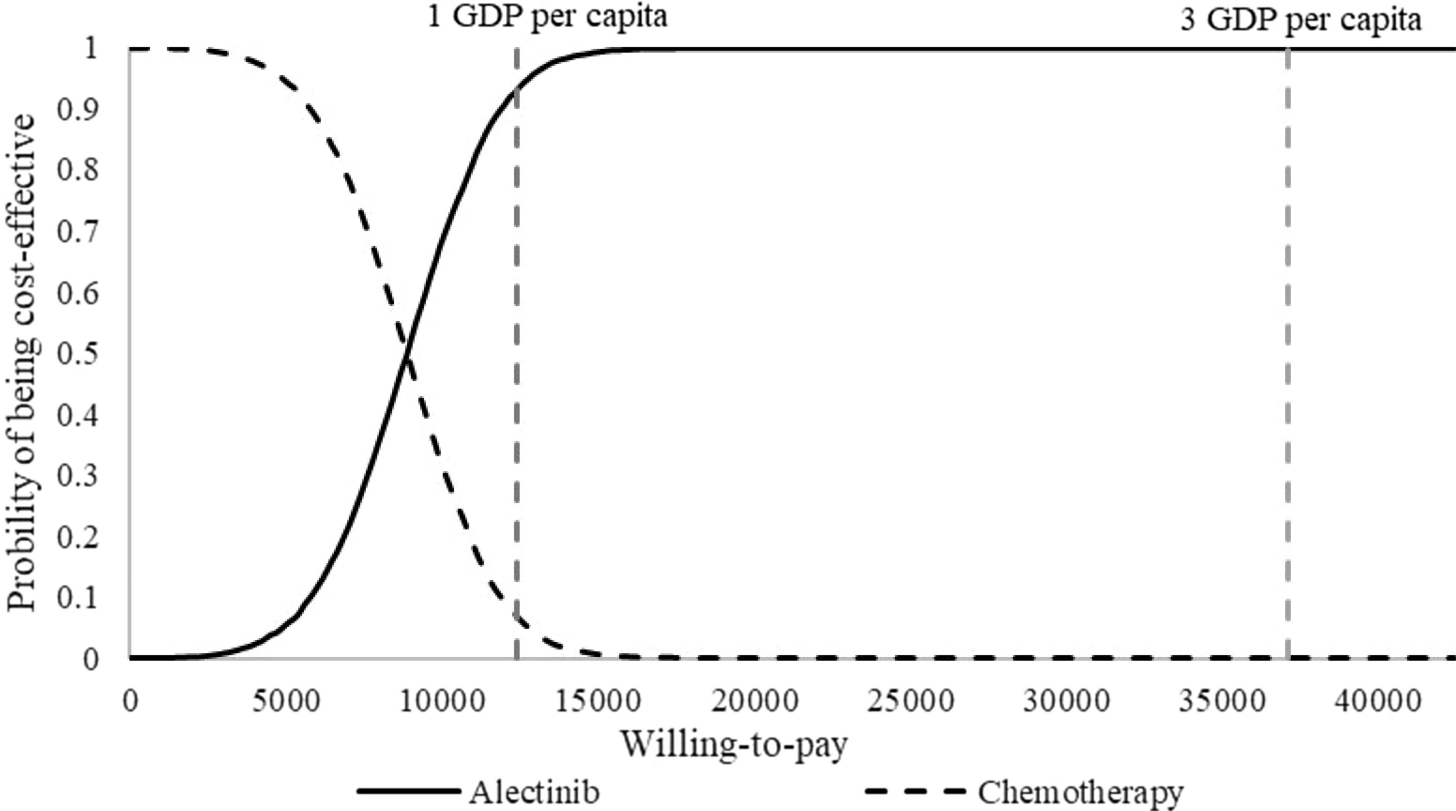
Cost‐effectiveness acceptability curves.

Cost‐effectiveness acceptability curves in Figure 4 illustrate the probability of each treatment being the more cost‐effective strategy at different WTP thresholds. Based on 5000 PSA iterations, alectinib had a 93% probability of being cost‐effective at a $12,367/QALY threshold (1 GDP per capita), and a 100% probability of being cost‐effective at a $37,100/QALY threshold (3 GDP per capita). Furthermore, a scatterplot in the cost‐effectiveness plane showed that all simulation results were in the northeast quadrant (see Figure S2). This indicated that alectinib was more effective than chemotherapy but also came with higher costs, similar to the base‐case ICERs. Overall, the results were robust in one‐way DSA and PSA.

### 3.3 Scenario Analyses Results

The results of scenario 1–3 are summarized in Table S4. In the first scenario, when the 1 L treatment regimen altered to lorlatinib, the incremental costs of the alectinib group decreased compared to chemotherapy, but minimal changes observed in health outcomes, resulting in a slight reduction in the ICER value. When the 1 L treatment regimen changed to alectinib, the incremental costs increased and the incremental health outcomes decreased, leading to a rise in the ICER value. In the second scenario, with a shortened time horizon, the ICERs of alectinib increased. ICERs of alectinib were $27,540/QALY, $11,664/QALY and $9276/QALY for the 10‐, 20‐, and 30‐year horizons, still remaining within 1–3 GDP per capita per QALY range. In the third scenario, when the proportion of patients actively receiving treatment was altered, the ICER of alectinib compared to chemotherapy decreased or increased slightly, but overall results were similar to those in the base‐case analyses.

## 4 Discussion

This is the first study to evaluate the cost‐effectiveness of adjuvant alectinib for the treatment of resected early‐stage ALK‐positive NSCLC. In this model‐based cost‐effectiveness analysis, alectinib increased total costs by $16,245 and provided gains of 2.02 LYs and 1.84 QALYs over a lifetime horizon, compared with platinum‐based chemotherapy. The ICERs were $8052/LY and $8806/QALY, respectively. Sensitivity and scenario analyses demonstrated the robustness of our results to variations in input parameters. The one‐way DSA found no threshold parameter was identified to change the base‐case cost‐effectiveness results at a threshold of $37,100/QALY. The results of probabilistic sensitivity analyses further showed that adjuvant alectinib was a cost‐effective option across 5000 Monte Carlo simulations. The probabilities of alectinib to be accepted as cost‐effective were 93% at $12,367/QALY threshold and 100% at $37,100/QALY threshold. Various scenarios, including 1 L treatment regimen in metastatic recurrence, time horizon, and proportion of patients actively receiving treatment, were explored to assess their impact on ICERs. Changing the 1 L treatment to either lorlatinib or alectinib did not significantly alter the base‐case analyses results. Shortening the time horizon notably increased ICERs. This was because the primary cost differences (specifically, the drug cost in DFS) were largely captured, while the health outcomes were not fully realized within these shorter horizons. Additionally, although ALK‐targeted therapies are now widely used in clinical practice according to expert opinions, a conservative estimate was conducted for the proportion of patients receiving active treatment. If the proportion of active treatment in each state were reduced, the ICERs increased.

A literature review on the economic evaluation of early‐stage NSCLC drug therapy was conducted and revealed that the health state involved multiple disease status such as DFS, disease progressed (including locoregional‐recurrence and metastatic recurrence), and death [34, 35, 36, 37, 38, 39]. Consistent with this finding, we employed a five‐state Markov model, specifically considering variation in cancer progression. In terms of cost and utility inputs, we primarily used data based on the Chinese population. This approach largely mitigated potential uncertainty arising from using cost and utility values from patients in other countries, given the economic and cultural differences between nations. Additionally, although clinical trials did not report the duration of AEs and detailed information on their treatment, we obtained relevant information through expert interviews and incorporated the costs and disutility of AEs into the model. The AEs profiles for these drugs should be a significant consideration when making individual treatment decisions.

Extrapolation methods were crucial in capturing the value of treatment regimens, considering the significant portion of clinical and economic benefits occurring outside the study duration [40]. Our study conducted survival estimation obtained from clinical trial data and compared various methods in terms of goodness‐of‐fit statistics to investigate the applicability of various extrapolation methods in cancer treatment. In addition, there were some key assumptions in our model to ensure that model inputs were aligned with real‐world practice. For example, we considered the possibility of cure, consistent with other cost‐effectiveness studies conducted in an adjuvant setting of NSCLC [34, 35, 36]. Besides, given the median follow‐up data from ALINE demonstrated that DFS benefit with alectinib was sustained for at least 28 months, our study assumed a conservative treatment effect duration for alectinib at 28–60 months. Of note, this assumption was similar to the recommendation from the National Institute for Healthcare and Excellence in the United Kingdom, concluding that a 3–5‐year treatment effect waning scenario is suitable to apply to the Generalized gamma, Gompertz and log‐normal progression‐free survival distributions [41]. To validate the accuracy of model analysis, we also compared the predicted survival curves with published long‐term data. Considering the relatively short time since the market release of alectinib, the chemotherapy group was selected as the comparison group. The ten‐year survival rate of the chemotherapy group was compared with long‐term data from Korean NSCLC patients who underwent pulmonary resection [42]. The ten‐year survival rate adjusted by the ALINA trial staging proportions in this study was 36.4%, lower than the ten‐year patient survival rate in our study (42.1%). The higher survival rate observed in our study may be attributed to advancements in medical care over time. Any future updates from the ALINE trial may provide the opportunity to further explore uncertainty regarding the curve extrapolation.

This study has several limitations that should be considered when interpreting the results. First, the limitation in accessing clinical data may affect the representation of benefits for the Chinese population. The ALINE trial included patients across 26 countries, with 55.6% from Asia. The lack of specific data for the Chinese subgroup could potentially impact the study results. Second, the ALINE trial did not provide detailed information on post‐nonmetastatic recurrence and metastatic recurrence treatment regimens. Due to the absence of relevant literature or databases, this study relied on published literature, clinical guidance and clinical experts to define the use of certain resources and estimate associated costs. Factors such as individual circumstances, past treatment history, and prescribing preferences of different hospital physicians can influence the real‐world selection of treatment regimens. Therefore, the information gathered from the above sources may not be comprehensive or objective, potentially leading to biased study results. Third, the model relied on various assumptions due to a lack of data to inform certain proportion and transition probabilities. While these assumptions were informed by clinical expert opinions and aligned with observations from the clinical trials used to inform model inputs, the model may not precisely depict the natural progression of the disease, particularly over the long term. Furthermore, given the lack of registry data, we relied solely on parametric models for curve extrapolation. This reliance on parametric models was also a significant source of uncertainty in our results. Fourth, the high number of early‐stage NSCLC patients cured through adjuvant treatment suggests potential productivity gains for both patients and caregivers. This aspect, often considered from a societal perspective, has not been considered in this analysis.

## 5 Conclusions

From the perspective of the health care system, alectinib appears to be the preferred cost‐effective option in the adjuvant treatment for Chinese patients with resected early‐stage ALK‐positive NSCLC. Our results may assist clinicians in making optimal treatment decisions for early‐stage NSCLC and offer value‐based evidence for drug access negotiations and pricing strategies within the Chinese healthcare system. Once long‐term study data is obtained, this study will conduct further analysis to validate the long‐term outcomes and reduce parameter uncertainty, thereby enhancing the quality of evaluation evidence and providing better support for decision making.

## Author Contributions

**Qiran Wei:** conceptualization (equal), data curation (equal), formal analysis (equal), investigation (equal), methodology (equal), project administration (equal), resources (lead), software (lead), supervision (equal), validation (equal), visualization (equal), writing – original draft (lead). **Yifang Liang:** data curation (equal), formal analysis (equal), investigation (equal), resources (equal), software (equal), writing – original draft (equal). **Jiahui Mao:** data curation (equal), formal analysis (equal), investigation (equal), resources (equal), software (equal). **Xin Guan:** conceptualization (lead), data curation (equal), formal analysis (equal), funding acquisition (equal), investigation (equal), methodology (equal), project administration (equal), resources (equal), supervision (equal), validation (equal), visualization (equal), writing – review and editing (lead).

## Ethics Statement

This study did not involve clinical results reporting.

## Conflicts of Interest

The authors declare no conflicts of interest.

## Supporting information


Data S1.

## Data Availability

The detailed information of parameters and their sources were performed in the tables and Supporting Information.
