# Supplementary material for: Cost‐Effectiveness Analysis of Adjuvant Alectinib versus Platinum‐Based Chemotherapy in Resected ALK‐Positive Non‐Small‐Cell Lung Cancer in the Chinese Health Care System
Source: Cancer Med. 2024 Nov 18;13(22):e70405. doi: 10.1002/cam4.70405 (PMC11571239; doi:10.1002/cam4.70405)
Supplement: Supplementary file 1 — Data S1. [file CAM4-13-e70405-s001.docx]

**Cost-effectiveness Analysis of Adjuvant Alectinib versus Platinum-based Chemotherapy in Resected ALK-Positive Non–Small-Cell Lung Cancer in the Chinese Health Care System**

**SUPPLEMENTS**

**
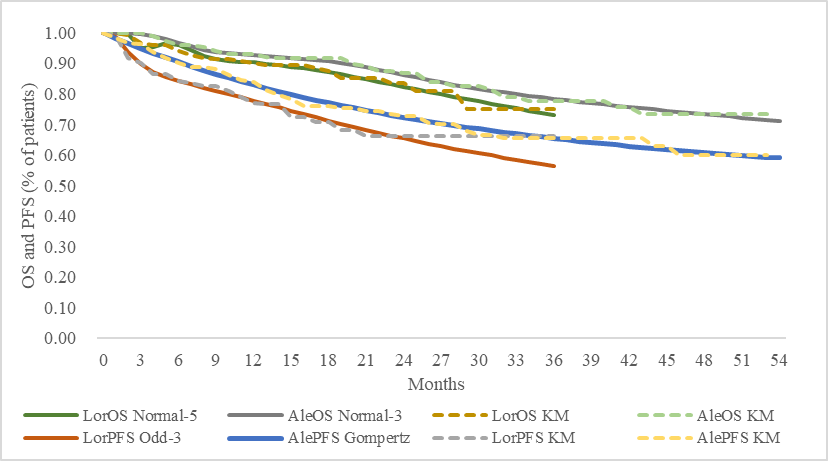
**

**Figure S1 KM and parametric survival distributions for the OS and PFS of lorlatinib and alectinib.** Lor, lorlatinib, OS, overall survival; Ale, alectinib; PFS, progression-free survival.

**
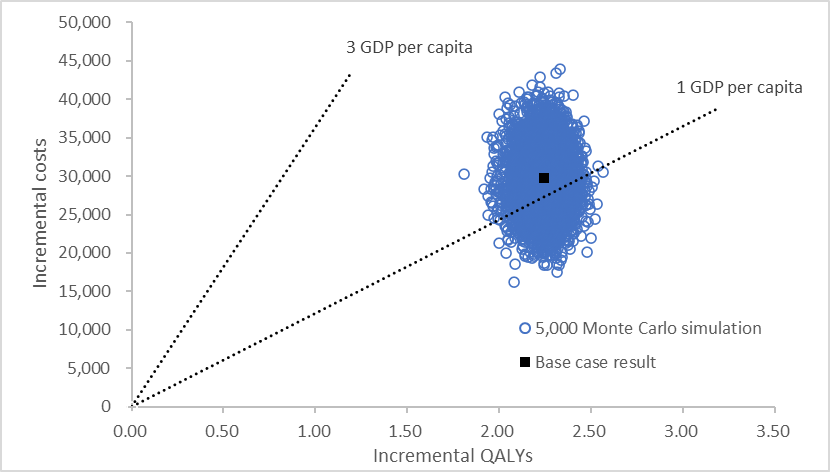
**

**Figure S2 A scatterplot in the cost-effectiveness plane.** QALY: quality-adjusted life-years.

**Table S1 CHEERS 2022 checklist.**

| **Topic** | **No.** | **Item** | **Location where items is reported** |
| --- | --- | --- | --- |
| **Title** |  |  |  |
|  | 1 | Identify the study as an economic evaluation and specify the interventions being compared. | Page1 Line1-3 |
| **Abstract** |  |  |  |
|  | 2 | Provide a structured summary that highlights context, key methods, results, and alternative analyses. | Page1-2 Line11-35 |
| **Introduction** |  |  |  |
| Background and objectives | 3 | Give the context for the study, the study question, and its practical relevance for decision making in policy or practice. | Page4-5 Line64-96 |
| **Methods** |  |  |  |
| Health economic analysis plan | 4 | Indicate whether a health economic analysis plan was developed and where available. | Not applicable |
| Study population | 5 | Describe characteristics of the study population (such as age range, demographics, socioeconomic, or clinical characteristics). | Page6-7 Line145-154 |
| Setting and location | 6 | Provide relevant contextual information that may influence findings. | Page5 Line103 |
| Comparators | 7 | Describe the interventions or strategies being compared and why chosen. | Page5-6 Line118-123 |
| Perspective | 8 | State the perspective(s) adopted by the study and why chosen. | Page5 Line112-113 |
| Time horizon | 9 | State the time horizon for the study and why appropriate. | Page5 Line105-106 |
| Discount rate | 10 | Report the discount rate(s) and reason chosen. | Page5 Line111 |
| Selection of outcomes | 11 | Describe what outcomes were used as the measure(s) of benefit(s) and harm(s). | Page5 Line108-111 |
| Measurement of outcomes | 12 | Describe how outcomes used to capture benefit(s) and harm(s) were measured. | Page5 Line108-111 |
| Valuation of outcomes | 13 | Describe the population and methods used to measure and value outcomes. | Page9-10 Line231-241 |
| Measurement and valuation of resources and costs | 14 | Describe how costs were valued. | Page8-9 Line205-229 |
| Currency, price date, and conversion | 15 | Report the dates of the estimated resource quantities and unit costs, plus the currency and year of conversion. | Page8-9 Line205-208 |
| Rationale and description of model | 16 | If modelling is used, describe in detail and why used. Report if the model is publicly available and where it can be accessed. | Page5 Line114-116 |
| Analytics and assumptions | 17 | Describe any methods for analysing or statistically transforming data, any extrapolation methods, and approaches for validating any model used. | Page5-6 Line114-143 |
| Characterising heterogeneity | 18 | Describe any methods used for estimating how the results of the study vary for subgroups. | Not applicable |
| Characterising distributional effects | 19 | Describe how impacts are distributed across different individuals or adjustments made to reflect priority populations. | Page11 Line266-277 |
| Characterising uncertainty | 20 | Describe methods to characterise any sources of uncertainty in the analysis. | Page10 Line243-264 |
| Approach to engagement with patients and others affected by the study | 21 | Describe any approaches to engage patients or service recipients, the general public, communities, or stakeholders (such as clinicians or payers) in the design of the study. | Not applicable |
| **Results** |  |  |  |
| Study parameters | 22 | Report all analytic inputs (such as values, ranges, references) including uncertainty or distributional assumptions. | Table1 |
| Summary of main results | 23 | Report the mean values for the main categories of costs and outcomes of interest and summarise them in the most appropriate overall measure. | Page11 Line280-287 |
| Effect of uncertainty | 24 | Describe how uncertainty about analytic judgments, inputs, or projections affect findings. Report the effect of choice of discount rate and time horizon, if applicable. | Page11 Line289-302 |
| Effect of engagement with patients and others affected by the study | 25 | Report on any difference patient/service recipient, general public, community, or stakeholder involvement made to the approach or findings of the study | Not applicable |
| **Discussion** |  |  |  |
| Study findings, limitations, generalisability, and current knowledge | 26 | Report key findings, limitations, ethical or equity considerations not captured, and how these could affect patients, policy, or practice. | Page12-15 Line315-394 |
| **Other relevant information** |  |  |  |
| Source of funding | 27 | Describe how the study was funded and any role of the funder in the identification, design, conduct, and reporting of the analysis | Page2 Line48 |
| Conflicts of interest | 28 | Report authors conflicts of interest according to journal or International Committee of Medical Journal Editors requirements. | Page2 Line50-51 |

*From:* *Husereau, Don et al. “Consolidated Health Economic Evaluation Reporting Standards (CHEERS) 2022 Explanation and Elaboration: A Report of the ISPOR CHEERS II Good Practices Task Force.” Value in health: the journal of the International Society for Pharmacoeconomics and Outcomes Research vol. 25,1 (2022): 10-31. doi:10.1016/j.jval.2021.10.008*

**Table S2 Results of AIC to the observed data in ITT population.**

|  | **AleDFS** | **ChemoDFS** | **LorOS** | **AleOS** | **LorPFS** | **AlePFS** |
| --- | --- | --- | --- | --- | --- | --- |
| Gamma | 199.0581 | 521.8486 | 271.6568 | 377.6071 | 428.1685 | 551.8287 |
| Gompertz | **197.5851** | 525.7885 | 271.6059 | 378.6438 | 424.9809 | **549.6371** |
| Weibull | 198.8743 | 522.7937 | 271.6668 | 377.7698 | 427.7233 | 551.4897 |
| Log-Logistic | 199.1960 | 520.0177 | 271.4986 | 377.3689 | 426.4576 | 550.4192 |
| Log-Normal | 201.2854 | **518.6793** | 270.2124 | 376.0274 | 423.6394 | 550.5034 |
| hazard3 | 202.1909 | 523.2148 | 271.9823 | 374.5460 | 423.8021 | 554.8395 |
| hazard4 | 204.7806 | 524.5204 | 272.6023 | 374.8114 | 424.1955 | 556.7767 |
| hazard5 | 203.9453 | 526.3825 | 268.1977 | 377.0046 | 425.8708 | 554.2227 |
| odd3 | 202.0136 | 523.2723 | 271.9759 | 374.5758 | **423.6231** | 554.8259 |
| odd4 | 205.8870 | 524.4082 | 272.5139 | 374.7874 | 424.0617 | 556.8290 |
| odd5 | 205.1804 | 526.3635 | 268.1040 | 377.0014 | 425.7303 | 554.0107 |
| nor3 | 201.7559 | 522.9007 | 271.5589 | **374.1916** | 424.2405 | 554.5625 |
| nor4 | / | 523.8341 | 271.8914 | 374.2318 | 423.8889 | 556.7021 |
| nor5 | 201.7727 | 525.9258 | **267.6447** | 376.5310 | 425.4169 | / |
| mixturegam | 201.0582 | 519.7272 |  |  |  |  |
| mixturegom | 199.5552 | 521.9175 |  |  |  |  |
| mixturewei | 200.8744 | 519.5840 |  |  |  |  |
| mixturellog | 201.1961 | 520.1308 |  |  |  |  |
| mixturelnorm | 203.2856 | 520.3386 |  |  |  |  |

AIC, Akaike information criterion; ITT, intention-to-treatment; Ale, alectinib; DFS, disease-free survival; Chemo, chemotherapy; Lor, lorlatinib, OS, overall survival; PFS, progression-free survival.

**Table S3: List of cost input parameters used in the Markov model.**

| **Cost inputs** | **Value ($)** | **Source** |
| --- | --- | --- |
| **Drug costs per cycle** |  |  |
| Alectinib | 1,937.93 | MENET |
| Cisplatin | 20.74-20.84 | MENET |
| Vinorelbine | 209.22 | MENET |
| Gemcitabine | 261.43 | MENET |
| Pemetrexed | 627.60 | MENET |
| Paclitaxel | 267.40-268.96 | MENET |
| Lorlatinib | 2,244.95 | MENET |
| Brigatinib | 1,697.85 | MENET |
| Bevacizumab | 4,658.64-4,667.27 | MENET |
| Sindillimab | 218.95 | MENET |
| **Follow-up costs per time or day** |  |  |
| Diagnosis | 1.42 | Health care document |
| Blood biochemistry tests | 42.56 | Health care document |
| Blood tests | 2.84 | Health care document |
| Urine analysis | 0.57 | Health care document |
| CT scans | 42.57 | Health care document |
| MRI scans | 60.31 | Health care document |
| **Administration costs per time** |  |  |
| Injection | 1.42 | Health care document |
| Nursing | 9.75 | Health care document |
| Monitoring | 5.96 | Health care document |
| Bed | 54.49 | Health care document |
| **AEs costs per time** |  |  |
| Blood creatine phosphokinase increased | 1.66 | Expert opinions, MENET |
| Neutrophil count decreased | 42.47 | Expert opinions, MENET |
| **Other costs per cycle** |  |  |
| Radiotherapy | 252.64 | Health care document |
| Best Supportive care | 350.64 | [1] |
| End of life | 1,967.49 | [2] |

CT: computed tomography; MRI: magnetic resonance imaging; AEs: adverse events.

**Table S4 Scenario analyses results.**

| **Scenario** | **Results** | **Ale** | **Chemo** | **Difference** |  |
| --- | --- | --- | --- | --- | --- |
| Scenario 1  (Using Ale as the 1L line treatment) | LYs | 10.74 | 8.22 | 2.52 |  |
|  | QALYs | 9.26 | 7.02 | 2.24 |  |
|  | Total cost ($) | 60,013 | 30,105 | 29,908 |  |
|  | ICER ($/LY) | 11,890 |  |  |  |
|  | ICER ($/QALY) | 13,376 |  |  |  |
| Scenario 2  (10-year horizon) | LYs | 6.88 | 5.81 | 1.07 |  |
|  | QALYs | 5.91 | 4.93 | 0.98 |  |
|  | Total cost ($) | 55,880 | 26,579 | 29,301 |  |
|  | ICER ($/LY) | 27,385 |  |  |  |
|  | ICER ($/QALY) | 29,990 |  |  |  |
| Scenario 2  (20-year horizon) | LYs | 9.43 | 7.41 | 2.02 |  |
|  | QALYs | 8.11 | 6.31 | 1.80 |  |
|  | Total cost ($) | 59,709 | 30,111 | 29,598 |  |
|  | ICER ($/LY) | 14,682 |  |  |  |
|  | ICER ($/QALY) | 16,427 |  |  |  |
| Scenario 2  (30-year horizon) | LYs | 10.46 | 8.04 | 2.42 |  |
|  | QALYs | 9.01 | 6.85 | 2.16 |  |
|  | Total cost ($) | 60,059 | 30,333 | 29,725 |  |
|  | ICER ($/LY) | 12,271 |  |  |  |
|  | ICER ($/QALY) | 13,786 |  |  |  |
| Scenario 3  (Setting proportion of patients receiving active treatment as 0.9, 0.8, 0.5) | LYs | 10.97 | 8.46 | 2.51 |  |
|  | QALYs | 9.45 | 7.22 | 2.23 |  |
|  | Total cost ($) | 63,521 | 34,367 | 29,154 |  |
|  | ICER ($/LY) | 11,618 |  |  |  |
|  | ICER ($/QALY) | 13,072 |  |  |  |
| Scenario 3  (Setting proportion of patients receiving active treatment as 0.8, 0.6, 0.4) | LYs | 10.47 | 7.71 | 2.76 |  |
|  | QALYs | 9.04 | 6.61 | 2.43 |  |
|  | Total cost ($) | 53,324 | 17,773 | 35,551 |  |
|  | ICER ($/LY) | 12,886 |  |  |  |
|  | ICER ($/QALY) | 14,636 |  |  |  |

Ale, alectinib; Chemo, chemotherapy; LY, life-year; ICER: incremental cost-effectiveness ratio; QALY: quality-adjusted life-year.

**References**

[1]. Xiang, G., et al., Economic Evaluation of First-Line Camrelizumab for Advanced Non-small-cell Lung Cancer in China. Front Public Health, 2021. 9: p. 743558.

[2]. Cao, H., J. Wang and Y. Shi, Investigation on medical expenses of patients with advanced malignant tumor in registered hospice care institutions in five provinces and cities. China General Practice, 2010. 13(31): p. 3544-3546.
